# Corrigendum

**DOI:** 10.1002/ehf2.12476

**Published:** 2019-06-19

In the paper by Masterson Creber *et al*.,^1^ there were some errors in *Table* 1.

The correct table is reproduced as follows:

**Table 1:** Palliative Performance Scale (PPSv2) version 2

PPS level (%)	Ambulation	Activity and evidence of disease	Self‐care	Intake	Conscious level
100	Full	Normal activity and work	Full	Normal	Full
		No evidence of disease			
90	Full	Normal activity and work	Full	Normal	Full
		Some evidence of disease			
80	Full	Normal activity with effort	Full	Normal or reduced	Full
		Some evidence of disease			
70	Reduced	Unable to do normal job/work	Full	Normal or reduced	Full
		Significant disease			
60	Reduced	Unable to do hobby/house work	Occasional assistance necessary	Normal or reduced	Full or confusion
		Significant disease			
50	Mainly sit/lie	Unable to do any work	Considerable assistance required	Normal or reduced	Full or confusion
		Extensive disease			
40	Mainly in bed	Unable to do most activity	Mainly assistance	Normal or reduced	Full or drowsy
		Extensive disease			+/− Confusion
30	Totally bed bound	Unable to do any activity	Total care	Normal or reduced	Full or drowsy
		Extensive disease			+/− Confusion
20	Totally bed bound	Unable to do any activity	Total care	Minimal to sips	Full or drowsy
		Extensive disease			+/− Confusion
10	Totally bed bound	Unable to do any activity	Total care	Mouth care only	Drowsy or coma
		Extensive disease			+/− Confusion
0	Death	—	—	—	—
